# Commentary on: The Gargano Yin Yang Breast Reduction Technique: How to Obtain Better Breast Shape, Volume Distribution, and Size With Long-Lasting Results

**DOI:** 10.1093/asjof/ojae125

**Published:** 2024-12-17

**Authors:** Elizabeth J Hall-Findlay

See the Original Article here.

Dr Francesco Gargano performs breast reduction using a medially based pedicle and an inferior flap to help reshape the breast.^1^ He says that “the aim of this article is to describe step by step a revolutionary breast reduction technique, giving a rationale for how each single surgical step may contribute to achieving what I consider, the ‘ideal breast.’” He followed 185 consecutive patients between 2001 and 2023, utilizing a “geometric model” to obtain a better shape.

Unfortunately, it is a bit misleading to say that the technique is “revolutionary.” There are numerous different techniques used to give good breast reduction results, and all techniques have added and adapted techniques from previous surgeons (just as Gargano has from both Tessier^2^ and Lejour^3^).

I agree with him that the BreastQ^4^ does not evaluate the aesthetic breast results and that we still need to define the “ideal” breast shape. This paper, however, does not provide any proof that this is a better method. He states that his paper is “evidence based,” but I do not see any evidence except measurements of the vertical incision length in 43 patients with a Wise pattern skin resection. He believes that pulling the inferior lateral flap medially will help reduce the base diameter of the breast, and he believes that suturing breast tissue to the pectoralis muscle will hold the breast up. Neither procedure is particularly effective.

**Figure 1.**
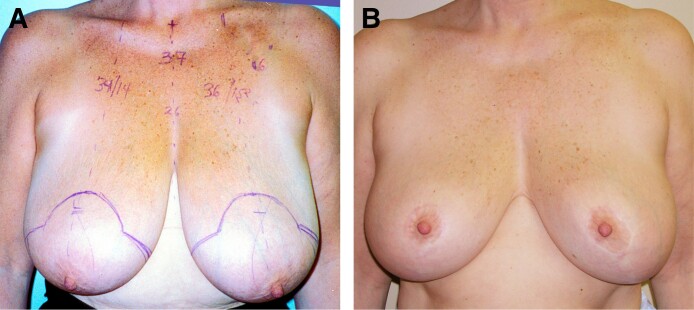
(A) A 47-year-old female patient underwent a breast reduction using a lateral pedicle. She had 370 g removed from the right breast and 425 g from the left breast. (B) She is shown at 8 years postoperatively with recurrence of the lateral fullness.

I tried a lateral pedicle in 224 patients because I thought it would have better sensation (it didn’t)^5^ and that rotating the pedicle medially would help to reduce the lateral fullness (it didn’t). Tension wins, and patients ended up with too much lateral fullness (Figure 1). This problem can be seen in Figure 9 in this article where the breast, despite the author's assertions, has a “boxy” shape with too much lateral fullness. Unfortunately, dermis (and to a lesser extent, parenchyma) stretches under tension.

I tried suturing breast tissue up to the pectoralis fascia in 77 patients (43 with absorbable sutures and 34 with permanent sutures). I managed to get 72 patients back in follow-up and in none of those patients did the elevation of the upper breast border last >5 months.^5^ Suturing breast tissue up to pectoralis fascia will not last (in contrast to Rubin's method,^6^ where he sutures the breast to the second rib). If the breast tissue permanently healed to pectoralis fascia, there would be a postoperative animation deformity.

**Figure 2.**
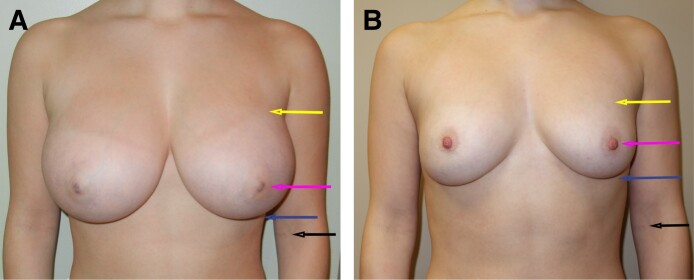
(A) A 17-year-old female patient underwent a breast reduction with removal of the posterior gland. No skin was removed. (B) She is shown at 1 year after surgery, and this result has been stable for over 10 years. The result was achieved with weight removal only. Note how the inframammary fold rose, but the upper breast border (yellow arrow) remained the same. Good results can be achieved with removal of weight and avoidance of tension. The nipple level (pink arrow) and the lower border of the breast (blue arrow) all rose compared to the elbow crease (black arrow) with removal of weight alone.

I also agree with Gargano that we “cannot rely on the skin brassiere alone to give shape and stability.” He believes instead in an “internal glandular brassiere” and an “internal support bra.” I believe instead that the key to long-lasting results is to remove the breast tissue where it is in excess—usually both inferior and lateral. I completely agree that it is important to reshape the gland and not rely on the skin as a brassiere, but I do not believe that we can rely on tension on the pillars to hold the shape, and I do not believe that sutures to the chest wall give a lasting result. Reshaping only works if the excess weight is removed. In fact, weight removal alone can give an excellent result (Figure 2).

Gargano notes that the inframammary fold (IMF) will rise with his technique of suturing the Wise pattern up to the chest wall. I do not perform any IMF sutures with the vertical approach and yet the IMF still rises. I have shown that this elevation results from weight removal alone and suturing the inferior breast down to the chest wall is unnecessary.

**Figure 3.**
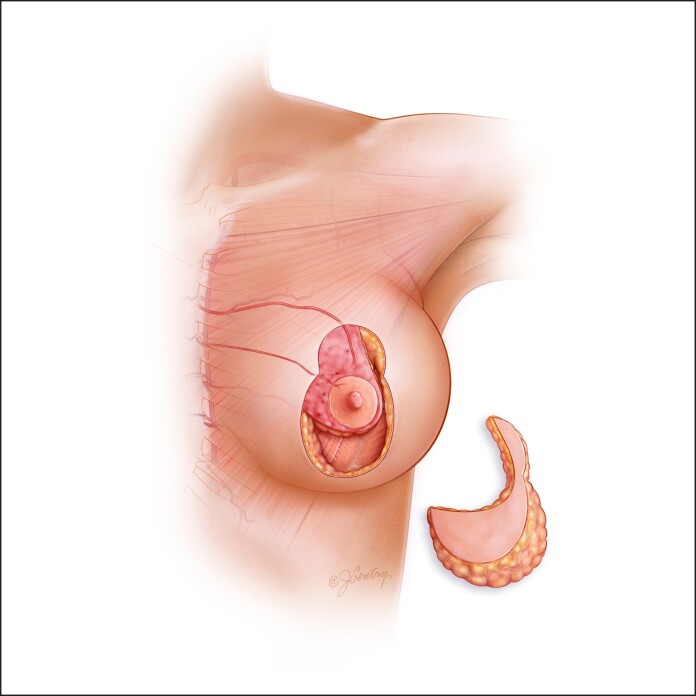
A true superomedial pedicle is safer than a pure medial pedicle because it has a dual axial blood supply. The descending artery from the second internal mammary webspace is a very strong vessel. It is more difficult to inset, but the parenchyma can be debulked because there is no blood supply through the parenchyma to any of the superiorly based pedicles.

Gargano is using a “medial” pedicle, not a true “superomedial” pedicle.^7^ The descending artery from the second interspace is a strong artery, and it should be included in a true superomedial pedicle by carrying the base of the pedicle lateral to the breast meridian (Figure 3). Relying on the vessels from the septum (fourth interspace) is not only unnecessary but can prevent adequate resection of the heavy inferior breast tissue. The blood supply to any of the superiorly based pedicles is superficial and does not travel through the parenchyma; therefore, the parenchyma can be debulked for an easier inset.

**Figure 4.**
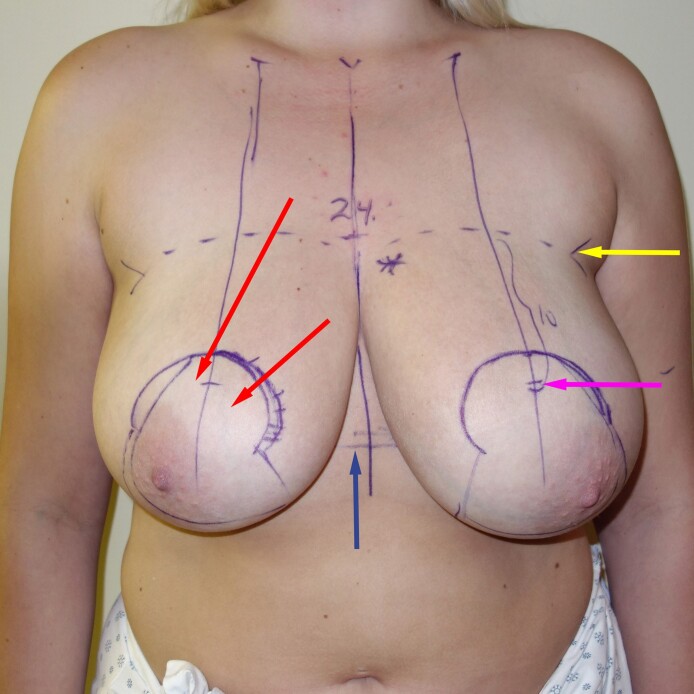
The preoperative markings in this 23-year-old female show that the inframammary fold is not as good a landmark as the upper breast border. Sometimes the breast footprint is so narrow vertically that the nipple would be placed too high. In this case, the breast footprint is so long vertically that the nipple would be placed too low. The upper breast border does not change in a breast reduction, and the ideal nipple position is 8 to 10 cm below the upper breast border.

The pedicle in this article is drawn differently in Figures 1 and 2, and the drawing in Figure 1 is far too high on the chest wall. Using that design will end up in nipples that are too high. The author states that the new nipple position should be 2 cm lower than the anterior projection of the IMF. Although I agree with not placing it too high, I believe that the IMF level can be misleading. Because the upper breast border does not change in a breast reduction, it is a more reliable landmark^8^ (Figure 4).

I would warn the author that placing his name on a technique can be fraught with misunderstanding by other surgeons. For example, I have never placed my name on my approach (which of course has been modified both by me and others over the years), yet the author states, “the aim of the Yin Yang technique is to avoid the pitfalls of the medial pedicle breast technique, as described by Hall-Findlay.”

**Figure 5.**
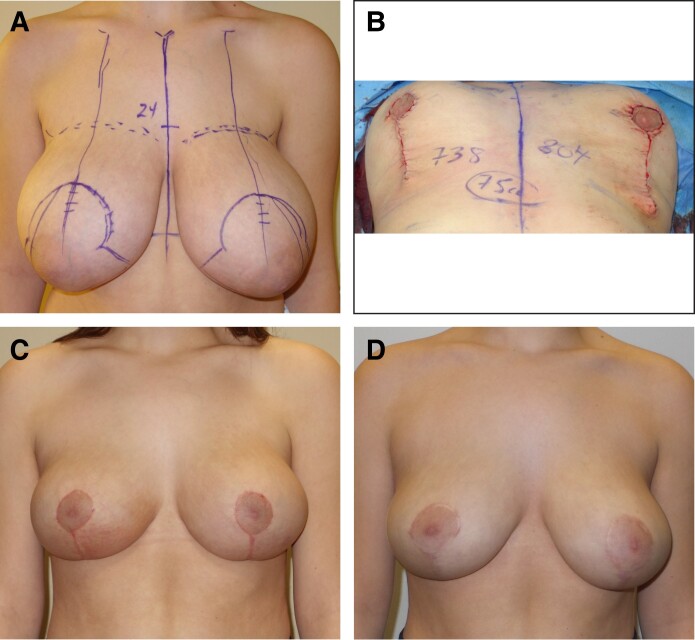
“Unsatisfactory results with the medial pedicle vertical breast reduction” are not the fault of the procedure but the fault of the surgeon. (A) This 16-year-old female patient underwent a vertical breast reduction using a true superomedial pedicle with 738 g removed from the right breast and 804 g removed from the left breast. (B) Intraoperatively, there are no sutures holding down the inframammary fold. (C) The patient is shown at 6 weeks postoperatively. The results do not need to look “unsatisfactory” in the early postoperative period. (D) The patient is shown at 1 year postoperatively. The asymmetry with the left breast being larger was my fault for not resecting enough weight. Comparing both sides shows that it was my fault, not the fault of the procedure.

He also states, “the unsatisfactory results attributable to the medial pedicle technique are intrinsic to the technique and have been documented by kinetic changes occurring in the immediate postoperative period, the major changes in terms of pseudoptosis happening in the first 12 months postoperatively.” Just as with this author, surgeons have criticized my vertical breast reduction procedure because they are not performing it the same way that I do. I do not cinch up the vertical incision (it doesn’t work)^9^ and I do not leave the breast shape concave inferiorly, leaving it to settle over time (Figure 5). “Pseudoptosis” or “bottoming-out” does not occur if the procedure is performed correctly. Too much postoperative lower pole excess is not a fault of the procedure but the fault of the surgeon who has performed an inadequate resection.

Gargano has developed an innovative variation for breast reduction surgery, but instead of trying to pull the lower pole of the breast and trying to hold parenchyma up with sutures, I think that he would get better results by respecting the role of weight and avoiding the effects of gravity instead.
